# A retrospective cohort study on impact of Covid-19 outbreak on out-of-hospital cardiac arrest (OHCA) in Telangana, India: mortality trends, contributing factors, and research imperatives

**DOI:** 10.1186/s12889-025-23917-4

**Published:** 2025-08-21

**Authors:** Amreen Aijaz Husain, Uddipak Rai, Amlan Kanti Sarkar, V. Chandrasekhar, Aditya Gupta

**Affiliations:** 1 School of Pharmaceutical and Population Health Informatics, DIT University, Dehradun, 248009 India; 2 Institution of Clinical Research India (ICRI), Mumbai, 400093 India; 3 Mahatma Gandhi Memorial Hospital, Warangal, 506002 India; 4 Department of Information and Communication Technology, University of Agder, Grimstad, 4886 Norway

**Keywords:** Cardiovascular diseases, Cardiopulmonary resuscitation, Emergency department, Emergency medical services, Out-of-hospital cardiac arrest, Return of spontaneous circulation

## Abstract

**Background:**

Out-of-hospital cardiac arrest (OHCA) poses considerable issues, notably in the context of the COVID-19 outbreak. The objective of this study is to assess the differences in incidence rates and clinical outcomes of OHCA in Telangana, India, between the pre-pandemic, pandemic, and post-pandemic periods, focusing on factors that influence survival rate.

**Methods:**

A retrospective cohort analysis was performed on patient records from a Telangana district hospital of those who presented to hospitals after OHCA from 2019 to 2021. The data were analysed to determine OHCA outcomes and factors that influence survival. Descriptive statistics, including frequencies and percentages, were used to summarise the categorical variables. The data were analysed to assess a significance level of *p* < 0.05. Appropriate statistical. methods, such as chi-squared and Fisher’s exact tests, were used to analyse quantitative variables. The 95% confidence interval (CI) was determined to analyse the precise distribution of variables across three time periods.

**Results:**

The number of OHCAs increased during the pandemic. The first wave accounted for 1,452 cases (31.34%), and the second accounted for 1,368 cases (29.53%), whereas there were 1,284 cases (27.72%) during the pre-pandemic period. The initiation rate of CPR dropped from 81.2% before the pandemic to 30.19% during the first wave and 49.02% during the second wave. Bystander-initiated CPR declined significantly. CPR was performed by laypersons in just 3.94% of cases during the first wave compared to 8.42% pre-pandemic. The time from OHCA to initiation of CPR was also significantly prolonged, with > 20-minute delays increasing from 15 to 80.3% during the first wave. ROSC rates remained tremendously low across all periods, with survival noted in just 0.26% of cases pre-pandemic and 0% across both waves of the pandemic. These findings highlight the severe impact of delayed emergency response and limited access to advanced care, as evidenced by the lack of ROSC during the epidemic.

**Conclusion:**

These. results demonstrate the value of early CPR and trained bystanders in the outcomes of OHCA. Individuals without this information should also be made aware of the ongoing need for public awareness and continued training in CPR, rapid EMS response times, and greater use of AEDs, especially in the context of revised guidelines and recommendations during the COVID-19 pandemic in 2020, to ensure that survival rates in such cases are improved.

## Summary

The COVID-19 pandemic affected patient outcomes, response times, and the incidence of OHCA care significantly [1]. Lockdowns, overwhelmed hospitals, and concern of COVID-19 exposure led to an increased incidence of OHCA as many individuals delayed seeking care for cardiovascular complaints [2]. The virus raised the risk of cardiac events outside of hospitals, as the virus itself represented cardiovascular threats [3]. Unprecedented challenges were raised for the emergency medical services (EMS) from high call numbers and new safety procedures (e.g., use of personal protective equipment [PPE]) that extended response times [4]. Concerns about viral transmission also led to lower bystander intervention rates, which reduced the chances of lifesaving early bystander cardiopulmonary resuscitation (CPR), an indispensable factor influencing OHCA survival [5]. This delay and alterations in EMS protocols led to poorer neurological outcomes and survival rates of OHCA patients during the pandemic [6]. Many hospitals were unable to provide optimal post-resuscitation care due to their engagement with COVID-19 patients. To address safety issues, modified CPR guidelines restricted some EMS techniques and recommended that bystanders perform hands-only CPR [4]. These changes posed an additional challenge to traditional resuscitative measures [7]. The pandemic highlighted the importance of community-trained CPR, the availability of automatic external defibrillators (AED), and flexible health policy to cover the need to provide emergency care in crisis-like situations [8]. Future readiness initiatives may help ensure that emergency response systems are more resilient and capable of providing critical care access in the event of public health emergencies, informed by the pandemic’s impact on OHCA [9].

##  Introduction

Out-of-hospital cardiac arrest (OHCA) is a critical and growing public health concern in India. OHCA is primarily associated with pre-existing cardiac conditions, which are on the rise due to urbanisation, lifestyle changes, and the increasing prevalence of risk factors such as hypertension, diabetes, tobacco use, and physical inactivity [10]. Consequently, sudden cardiac arrest events are becoming more frequent, often leading to emergency department visits with limited opportunities for timely intervention [11]. Epidemiological data on OHCA in India are scarce, and survival rates remain significantly lower than in high-income countries due to the absence of a well-established prehospital emergency care system [12]. While national data remain limited, existing reports indicate alarmingly high OHCA incidence and mortality rates [13]. In many regions, emergency medical services (EMS) are either underdeveloped or face substantial response delays [14]. Additionally, bystander intervention is hindered by a lack of CPR training and awareness, while automatic external defibrillators (AEDs) are not widely available, even in high-risk public spaces [15]. Geographic diversity and healthcare disparities further challenge the timely delivery of emergency care [16]. Although metropolitan areas may have relatively modern EMS infrastructure, vast rural and semi-urban regions of India’s population still lack an organised emergency response system [17]. These gaps contribute to poor OHCA survival rates, as delays in medical intervention significantly reduce chances of survival [18]. Efforts are being made to increase public awareness, enhance EMS accessibility, and expand CPR training programs [17, 18]. The establishment of AED networks in public spaces and the development of standardised OHCA response systems are significant milestones. However, overcoming these challenges requires a coordinated approach involving government support, healthcare organisations, and community engagement [19]. Strengthening India’s emergency care framework, alongside public education and advocacy, is crucial in reducing the burden of OHCA and improving survival outcomes nationwide [20]. The COVID-19 pandemic, caused by severe acute respiratory syndrome coronavirus 2 (SARS-CoV-2), was first reported in China in December 2019, with India confirming its first case in Kerala on January 27, 2020. The World Health Organisation (WHO) declared COVID-19 a global pandemic on March 11, 2020. India imposed a nationwide lockdown starting March 25, 2020, with Kerala initiating restrictions earlier on March 23. The first wave extended from March 2020 to January 2021, whereas the second wave, beginning in March-April 2021, was shorter but led to a rapid rise in cases and mortality over the following months [21, 22]. Beyond direct COVID-19-related deaths, concerns grew regarding its impact on emergency healthcare services, including OHCA management [23]. Lockdowns and movement restrictions, coupled with public fear of hospital contamination, led to delays in EMS activation and emergency room visits, exacerbating treatment delays and reducing the appropriateness of medical interventions [24]. Overall, the indirect effects of the pandemic posed significant threats to public health [25]. OHCA serves as a key indicator of both population health and the healthcare system’s ability to manage emergencies [26]. COVID-19 can induce acute respiratory distress syndrome (ARDS) and excessive immune responses, which can be fatal [27]. Consequently, some of the OHCAs recorded during the pandemic may have resulted from undiagnosed respiratory complications in non-hospitalized COVID-19 patients [28]. Further raising the risk of OHCA, COVID-19 has also been linked to acute cardiovascular events like myocarditis, arrhythmias, acute coronary syndromes, and heart failure. Experimental treatments like hydroxychloroquine and azithromycin may have also contributed to cardiac events [29]. Limited medical resources during the pandemic led to hospital admission prioritisation, which may have resulted in more OHCA cases occurring at home [30]. Psychological stress, restricted movement, and grief from pandemic-related losses may have further contributed to adverse cardiac events and OHCA incidents [31]. Given these factors, large-scale research is necessary to analyse OHCA trends, identify predictors, and implement strategies to improve outcomes. Such insights will be particularly beneficial for the Indian healthcare system [32]. While this study discusses the broader context of EMS access and bystander intervention to frame the public health significance of OHCA outcomes, its analytic focus is limited to patients who were brought alive or dead to the hospital. Field-level events, such as arrests or people never transported or declared dead in the field, were outside the scope of this analysis due to data unavailability in this regional EMS structure. This study was performed in Warangal, Telangana—a region characterised by a blend of urban and semi-urban demographics exhibiting notable discrepancies in healthcare availability. Mahatma Gandhi Memorial Hospital (MGMH), a data collection site, is a secondary care District Headquarters Hospital serving an annual population of around 2 million. The hospital functions as the principal referral facility for emergency cases in many adjacent districts in North Telangana. The emergency medical services (EMS) in this region are poorly developed, characterised by a lack of qualified paramedics, advanced life support (ALS) ambulances, and streamlined prehospital care systems. Family members or basic EMS services, which do not implement pre-arrival stabilising techniques, typically bring patients suffering from OHCA to hospital. These variables significantly affect the methodological framework of this retrospective analysis, which concentrated on evaluating patient outcomes upon admission at the emergency room, omitting prehospital survival metrics due to inconsistent or missing field-level data. In this local setting, the study attempts to fill gaps in OHCA epidemiology by assessing the impact of delays in EMS activation, CPR initiation, and having access to definitive care on survival outcomes during three phases of the COVID-19 pandemic. These understandings are essential for guiding regional public health strategy and enhancing future emergency care preparedness.

## Objective

The study aimed to compare the rates of OHCA cases and their immediate outcomes in the emergency department before and during the pandemic, focusing on documented CPR initiation, transport timelines, and early survival indicators.

### Methodology

The study design for the retrospective hospital Record-Based study is shown in Fig. 1

### Study design

**Fig. 1:**
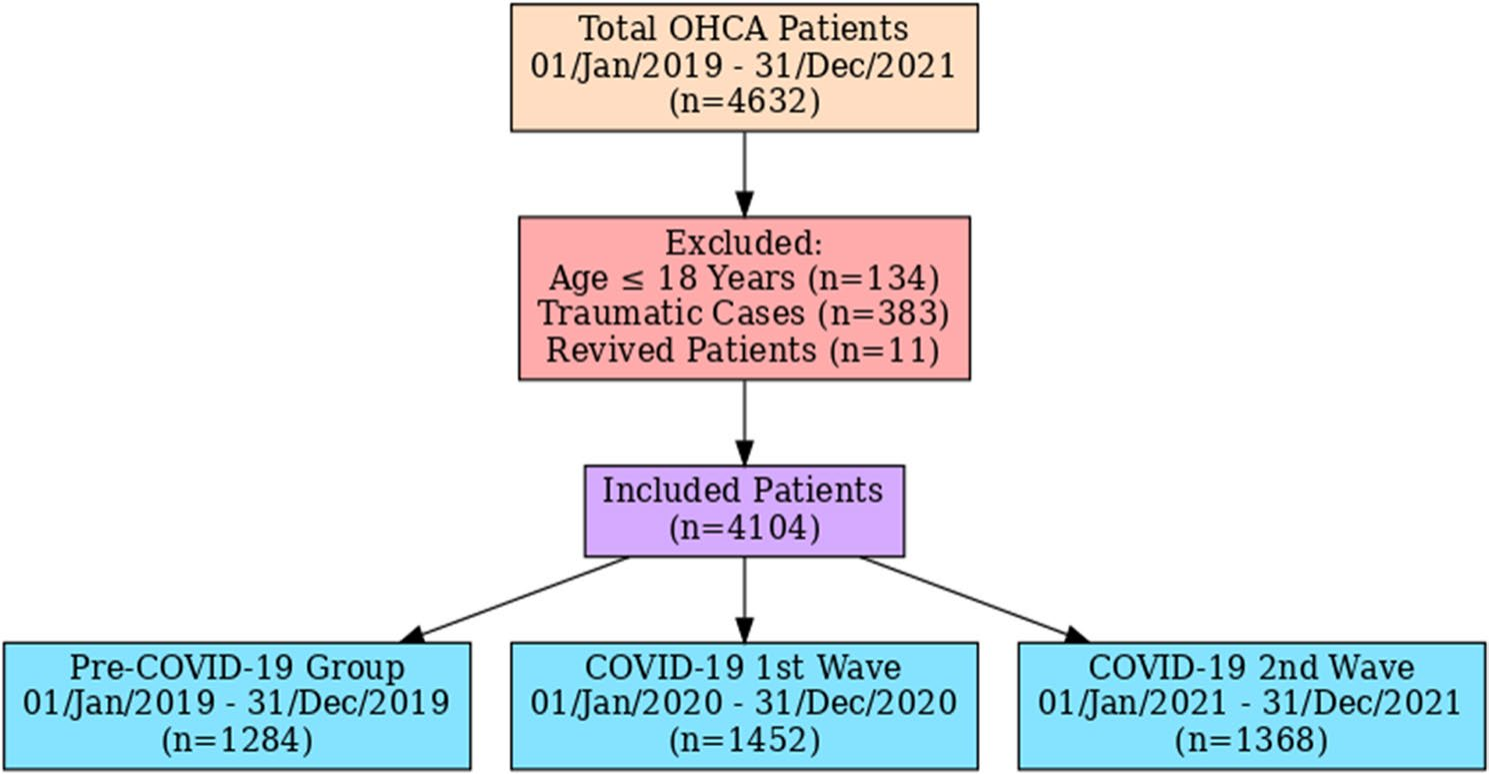
Flow diagram of study design

#### Study setting

This retrospective observational study utilised data from the emergency department of Mahatma Gandhi Memorial Hospital, a secondary care district headquarters hospital located in Warangal, Telangana. The hospital provides medical care to approximately 2,000,000 people annually in a low-resource setting and was the site for our study conducted from January 1, 2019, to December 31, 2021. The hospital receives high volumes of emergency cases, particularly OHCAs transported by their family members, private vehicles, or basic ambulance services. There is less developed EMS infrastructure in this area, with relatively few trained paramedics, ALS ambulances, or standardised prehospital care protocols.

We intend to reach out to all OHCA patients who were transported to and registered at the emergency department between the pre-pandemic and pandemic timelines. Patients declared dead or revived at the scene were not included, as the objective was to evaluate the effect of prehospital and early hospital care on patient outcomes, including survival to discharge and in-hospital mortality among patients arriving at the emergency department. The retrospective data of individual patients were collected from the MRD (Medical Record Section) of the Emergency Department.

#### Study participants

The study subjects included all adults over 18 years of age who were admitted to the hospital during the period of the study (from 01/January/2019 to 31/Dec/2021) and with a history of non-traumatic OHCA presumed to have cardiac aetiology throughout the duration of the study. This analysis only included OHCA cases that arrived at the hospital and registered in the emergency department. This study excluded arrests that were not taken to the hospital or were declared dead in the field. This omission was primarily due to the limited presence of trained EMS providers and field death certification protocols in the region, which typically results in nearly all suspected OHCA victims being brought to the hospital, regardless of viability.

- Sample Size: The total sample size included 4,104 subjects.
- Inclusion Criteria: All subjects (victims) of OHCA above 18 years.
- Exclusion Criteria:
  Subjects (victims) of OHCA whose data is unavailable or whose attendees are reluctant to provide the data are not included in the research.
  Subjects revived before coming to the hospital.
  Subjects with traumatic cardiac arrest are excluded, except for those who have experienced trauma, hanging, or burns

We performed specific subgroup analyses on smaller cohorts (500 participants or more), while the total sample size included 4,104 subjects. We make this distinction to ensure clarity in reporting the overall study population versus specific analyses of the subgroups.

#### Ethical consideration

This analysis was a retrospective cohort held at a single centre in the Mahatma Gandhi Memorial Secondary Care Healthcare Facility in Warangal, Telangana. The DIT University Research Ethics Committee (Protocol no. DITU/UREC/2022/04/4) and Institutional Ethics Committee KIEC, (Reference number: ECR/840/Inst/TG/2016/RR/20/29) provided approval for this study. The research adhered to the protocol while protecting the subjects’ (victims) right to privacy.

### Statistical analysis

We entered the data into a structured database using Microsoft Excel and MedCalc Statistical Software version 19.3.1, and utilised SPSS for analysis. Depending on whether the distribution is normal or non-normal, quantitative variables were summarised as either the mean S.D. and 95% C.I. of the mean or the median. Categorical variables were summarised using frequency, percentage, proportion, and the 95% confidence interval (CI) of the percentage. Appropriate statistical methods, such as chi-square and Fisher’s exact tests, were used to analyse quantitative variables. A *p*-value of under 0.05 is considered significant, and actual *p*-values are reported (Tables 1, 2 and 3) [33].

**Table 1:** Baseline data for OHCA subjects during pre-COVID-19 and COVID-19 (1st and 2nd wave) outbreak groups

S. No.	Variable (*N* = 4104)		Pre-Covid-19 (2019), (*n* = 1284)		First Wave (2020), (*n* = 1452)		Second Wave (2021), (*n* = 1368)		*P* value
			Frequency	Percentage (95% CI)	Frequency	Percentage (95% CI)	Frequency	Percentage (95% CI)	
**1**	Sex	Male	879	68.45% (65.92%, 71.00%)	1015	69.90% (67.54 − 72.26%)	943	68.93% (66.48 −71.39%)	0.586
		Female	405	31.54% (29.00 − 34.08%)	437	30.09% (27.74 − 32.46%)	425	31.06% (28.61 − 33.52%)	
**2**	Age (Years)	18–45	175	13.62% (11.86 − 15.61%)	192	13.22% (11.58 − 15.06%)	178	13.01% (11.33 − 14.90%)	0.912
		45–60	774	60.28% (57.58 − 62.92%)	892	61.43% (58.90 − 63.90%)	837	61.18% (58.57 − 63.73%)	
		> 60	335	26.09% (23.76 − 28.56%)	368	25.34% (23.17 − 27.64%)	353	25.80% (23.56 − 28.19%)	
**3**	Location of OHCA	Residence	1088	84.73% (82.66 − 86.60%)	1367	94.14% (92.82 − 95.24%)	1213	88.66% (86.88 − 90.24%)	< 0.001
		Public space	158	12.30% (10.62 − 14.22%)	56	3.85% (2.98 − 4.98%)	126	9.21% (7.79 − 10.86%)	
		Hospital (MGMH)	32	2.49% (1.77 − 3.50%)	24	1.65% (1.11 − 2.45%)	25	1.82% (1.24 − 2.68%)	
		Other healthcare area	6	0.46% (0.21 − 1.02%)	5	0.34% (0.15 − 0.80%)	4	0.29% (0.11 − 0.75%)	
**4**	Time of OHCA	Morning hours	567	44.15% (41.46 − 46.89%)	645	44.42% (41.88 − 46.99%)	639	46.71% (44.08 − 49.36%)	0.548
		Afternoon hours	364	28.34% (25.95 − 30.88%)	419	28.85% (26.58 − 31.24%)	384	28.07% (25.75 − 30.51%)	
		Evening and late-night hours	353	27.49% (25.12 − 30.00%)	388	26.72% (24.51 − 29.06%)	345	25.21% (22.99 − 27.59%)

**Table 2:** Clinical Characteristics of OHCA subjects during pre-COVID-19 and COVID-19 (1st and 2nd wave) outbreak groups

S. No.	Clinical Features		Pre-Covid-19 (2019)		First Wave (2020)		Second Wave (2021)		*P* Value
			Frequency	Percentage (95% CI)	Frequency	Percentage (95% CI)	Frequency	Percentage (95% CI)	
**1**	Risk factors	Yes	714	72.27% (69.48 −75.06%)	996	87.75% (85.84 − 89.66%)	860	81.52% (79.18 − 83.86%)	*p* < 0.001
	(*n* = 988, 1135, 1055)	No	274	27.73% (24.94 − 30.52%)	139	12.25% (10.34 − 14.16%)	195	18.48% (16.14 − 20.82%)	
**2**	Prior cardiac disease history	Yes	224	24.24% (21.48 − 27.00%)	268	25.24% (22.63 − 27.85%)	248	25.15% (22.44 − 27.86%)	0.912
	(*n* = 924, 1062, 986)	No	700	75.76% (73.00 − 78.52%)	794	74.76% (72.15 − 77.37%)	738	74.85% (72.14 − 77.56%)	
**3**	Comorbid illness	Cancer	27	8.44% (5.39 − 11.49%)	30	8.31% (5.46 − 11.16%)	29	8.66% (5.65 − 11.67%)	0.973
	(*n* = 320, 361, 335)	Respiratory disease	92	28.75% (23.79 − 33.71%)	184	50.97% (45.81 − 56.13%)	116	34.63% (29.53 − 39.73%)	< 0.001
		Renal disease	68	21.25% (16.77 − 25.73%)	76	21.05% (16.84 − 25.26%)	70	20.9% (16.55 − 25.25%)	0.988
		Neurologic	57	17.81% (13.62 − 22.00%)	62	17.17% (13.28 − 21.06%)	59	17.61% (13.53 − 21.69%)	0.963
		Thyroid disease	13	4.06% (1.90 − 6.22%)	15	4.16% (2.10 − 6.22%)	14	4.18% (2.04 − 6.32%)	0.996
		Liver disease	18	5.63% (3.10 − 8.16%)	21	5.82% (3.40 − 8.24%)	19	5.67% (3.19 − 8.15%)	0.981
		Other	45	14.06% (10.25 − 17.87%)	83	22.99% (18.65 − 27.33%)	58	17.31% (13.26 − 21.36%)	< 0.001
**4**	Preceding symptoms present	Yes	756	63.26% (60.53 − 65.99%)	754	55.65% (53.00 − 58.30%)	805	63.09% (60.44 − 65.74%)	< 0.001
	(*n* = 1195, 1355, 1276)	No	439	36.74% (34.01 − 39.47%)	601	44.35% (41.70 − 47.00%)	471	36.91% (34.26 − 39.56%)	
**5**	Symptom Duration	< 1 h	686	81.09% (78.45 − 83.73%)	795	81.96% (79.54 − 84.38%)	736	81.42% (78.88 − 83.96%)	0.935
	(*n* = 846, 970, 904)	1–24 h	117	13.83% (11.50 − 16.16%)	131	13.51% (11.36 − 15.66%)	129	14.27% (11.99 − 16.55%)	
		> 24 h	43	5.08% (3.60 − 6.56%)	45	4.64% (3.32 − 5.96%)	39	4.31% (2.99 − 5.63%)	

*p < 0.001: The *p*-value is extremely small (4.06 × 10^−9^) which is much less than the typical significance level of 0.05. This indicates a highly significant difference in the proportion of respiratory disease across the three groups.

The table investigates clinical variables related with OHCA cases over three time periods: pre-COVID-19 (2019), the first wave (2020), and the second wave (2021).

**Table 3:** Survival rate of OHCA subjects after resuscitation attempt during pre-COVID-19 and COVID-19 (1st and 2nd wave) outbreak groups

Features		Pre-Covid-19 (2019)		First Wave (2020)		Second Wave (2021)		*P* Value
		Frequency	Percentage	Frequency	Percentage	Frequency	Percentage	
1. OHCA witnessed (*n* = 978, 1115, 1039)	Yes	905	92.54% (90.89 - 94.19%)	427	38.3% (35.45 − 41.15%)	657	63.23% (60.30 − 66.16%)	< 0.001
	No	73	7.46% (5.82 − 9.11%)	588	52.74% (58.85 − 64.56%)	382	36.77 (33.83 − 39.70%)	
2. First responder witnessed (*n* = 933, 1057, 987)	Relative	919	98.5% (97.72 − 99.28%)	563	53.26% (50.25 − 56.27%)	628	63.63% (60.63 − 66.63%)	< 0.001
	Other	68	7.29% (5.62 − 8.96%)	494	46.74% (43.73 − 49.75%)	359	36.37% (33.37 − 39.37%)	
3. EMS called (*n* = 964, 1028, 971)	Yes	463	48.03% (44.88 − 51.18%)	802	78.02% (75.49 − 80.55%)	692	71.27% (68.42 − 74.12%)	< 0.001
	No	501	51.97% (48.82 − 55.12%)	226	21.98% (19.45 − 24.51%)	279	28.73% (25.88 − 31.58%)	
4. CPR initiated (*n* = 936, 1093, 1016)	Yes	760	81.2% (78.70 − 83.70%)	330	30.19% (27.47 − 32.91%)	498	49.02% (45.95 − 52.09%)	< 0.001
	No	176	18.8% (16.30 − 21.30%)	763	69.81% (67.09 − 72.53%)	518	50.98% (47.91 − 54.05%)	
5. CPR initiated by (*n* = 760, 330, 498) ***	EMS person	348	45.79% (42.25 − 49.33%)	162	49.09% (43.70 − 54.48%)	258	51.81% (47.42 − 56.20%)	< 0.001
	Trained personnel	312	41.05% (37.55 − 44.55%)	149	45.15% (39.78 − 50.52%)	206	41.37% (37.04 − 45.70%)	
	Lay person - family	64	8.42% (6.45 − 10.39%)	13	3.94% (1.84 − 6.04%)	21	4.22% (2.45 − 5.99%)	
	Lay person - Stranger	36	4.74% (3.23 − 6.25%)	6	1.82% (0.38 − 3.26%)	13	2.61% (1.21 − 4.01%)	
6. Time interval between OHCA and initiation of CPR (*n* = 760, 330, 498)	< 5 min	64	8.42% (6.45 − 10.39%)	7	2.12% (0.57 − 3.67%)	11	2.21% (0.92 − 3.50%)	< 0.001
	05–20 min	582	76.58% (73.57 − 79.59%)	58	17.58% (13.47 − 21.69%)	94	18.88% (15.44 − 22.32%)	
	> 20 min	114	15% (12.46 − 17.54%)	265	80.3% (76.01 − 84.59%)	393	78.92% (75.34 − 82.50%)	
7. Time of arrival of EMS (*n* = 964, 1028, 971) *****	< 10 min	42	4.36% (3.07 − 5.65%)	5	0.49% (0.06 − 0.92%)	9	0.93% (0.33 − 1.53%)	< 0.001
	10 to 30 min	911	94.5% (93.06 − 95.94%)	231	22.47% (19.92 − 25.02%)	568	58.5% (55.40 − 61.60%)	
	> 30 min	11	1.14% (0.47 − 1.81%)	792	77.04% (74.47 − 79.61%)	394	40.58% (37.49 − 43.67%)	
8. AED/Defibrillator used (*n* = 242, 349, 327)	Yes	97	40.08% (33.91 − 46.25%)	33	9.46% (6.39 − 12.53%)	71	21.71% (17.24 − 26.18%)	< 0.001
	No	145	59.92% (53.75 − 66.09%)	316	90.54% (87.47 − 93.61%)	256	78.29% (73.82 − 82.76%)	
9. ROSC achieved (Survived)	Yes	2	0.26% (− 0.10 − 0.62%)	0	0% (0.0% − 0.0%)	0	0% (0.0% − 0.0%)	0.068
(Not-survived) (*n* = 760, 330, 498)	No	758	99.74% (99.38 − 100.10%)	330	100% (100.0% − 100.0%)	498	100% (100.0% − 100.0%)	
10. Time interval between OHCA and death of the patient (*n* = 1284, 1452, 1368)	< 12 h	1212	94.39% (93.13 − 95.65%)	1197	82.44% (80.48 − 84.40%)	1290	94.3% (93.07 − 95.53%)	< 0.001
	12–24 h	64	4.98% (3.79 − 6.17%)	252	17.36% (15.41 − 19.31%)	69	5.04% (3.88 − 6.20%)	
	> 24 h	8	0.62% (0.19 − 1.05%)	3	0.21 (− 0.03 − 0.45%)	9	0.66 (0.23 − 1.09%)	

The chart shows a comprehensive overview of OHCA characteristics and reactions before COVID-19 (2019), the First Wave (2020), and the Second Wave (2021). The following is a study of important trends and insights

The study data were analysed using univariate methods due to collinearity concerns or the observational nature of the study.

### Data sources and data collection

Those over the age of 18 admitted to the hospital with a history of non-traumatic OHCA of suspected cardiac origin during the COVID-19 pandemic were included in the study. The geographic area was chosen as a sample of Telangana’s communities, with similar levels of awareness and access to healthcare across the state. Patients with OHCA who presented to the ED in the community or at home were given CPR according to advanced cardiovascular life support (ACLS) guidelines. Participants were enrolled in the study whether they were reported dead on arrival or if they survived/were revived after CPR after OHCA.

We obtained data on OHCA from the hospital’s emergency records and the Medical Records Department (MRD) using a validated OHCA data collection form. In this area, emergency medical services are restricted, and ACLS is predominantly inaccessible. The majority of cardiac arrest patients are transported to the hospital by either family members or minimal ambulance services, which lack on-site medical evaluation or stabilisation. “Dead on arrival” (DOA) refers to patients who reach the hospital with no vital signs and are pronounced deceased quickly after evaluation by the emergency medical officer. In this context, EMS infrequently issues death declarations in the field due to restricted training and legal limitations. As a result, almost all presumed OHCA sufferers are brought to the hospital, irrespective of whether the cardiac arrest happened minutes or hours prior. This study does not account for field-based terminations of resuscitation or arrests that were not transported to the hospital. We obtained missing data or further information by telephone from the victim’s family and attendants. The data was collected in accordance with local legal and ethical requirements. Retrospective data was collected manually from the casualty emergency death register records.

## Results

During the study period, 4632 total cases of OHCA were documented. Out of which 4104 cases (1284 in 2019, 1452 in 2020, and 1368 in 2021) were eligible for inclusion in the analysis after the exclusion of the following cases: Age: ≤ 18 years (*N* = 134), traumatic cardiac arrest (*N* = 383), and revived patients (*N* = 11), illustrated in Fig. 1. The incidence of OHCA increased during the pandemic. During the first wave, 1452 cases were recorded, accounting for 31.34% of the total cases, while the second wave reported 1368 cases (29.53%). In comparison, the pre-pandemic period recorded 1,284 cases (27.72%). This increase during the pandemic reflects the heightened strain on healthcare systems and the potential indirect effects of COVID-19 on cardiac health.

The findings demonstrate the pandemic’s impact on OHCA incidence trends and demographic features of OHCA cases over three time periods: pre-COVID-19 (2019), the first wave (2020), and the second wave (2021). 1.

### Sex distribution

**Fig. 2:**
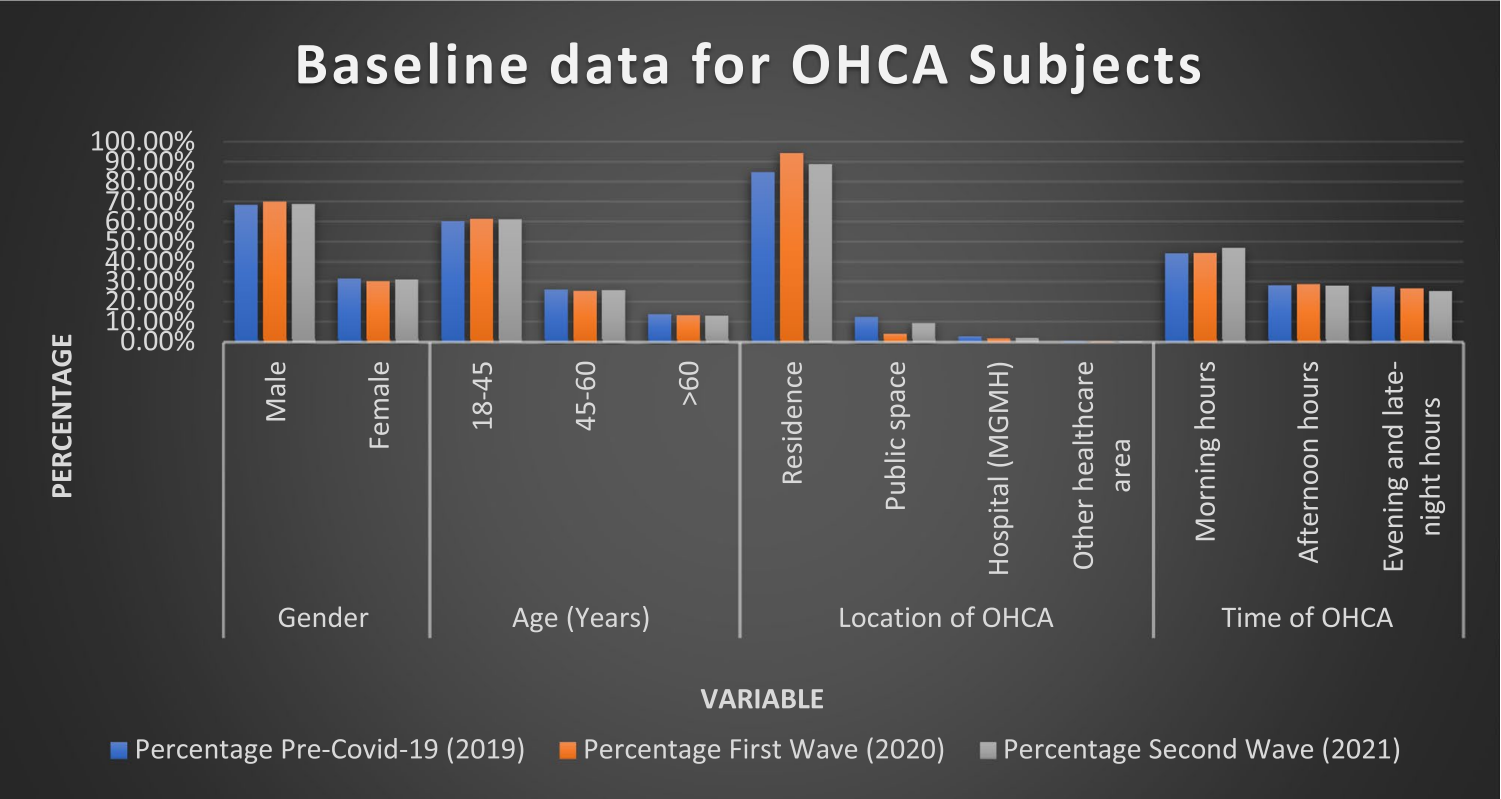
Baseline data for OHCA subjects during pre-COVID-19 and COVID-19 (1st and 2nd wave) outbreak groups

Table 1; Fig. 2 illustrated that males accounted for the majority of OHCA cases in 2019, 2020, and 2021 (68.45%, 69.90%, and 68.93%, respectively). Female cases demonstrated comparable stability, ranging from 30.09 to 31.54%. The lack of significant variations suggests that the pandemic had minimal impact on OHCA incidence based on sex.

### Age distribution

Individuals aged 45–60 reported approximately 60% of OHCAs across all time periods. This trend continued consistently, with percentages of 60.28% in 2019, 61.43% in 2020, and 61.18% in 2021, as shown in Table 1; Fig. 2. Approximately 25% of instances were reported by people aged > 60, with those between ages 18 and 45 accounting for only about 13%. The steady age distribution shows that the pandemic had no significant impact on the demographic profile of OHCA patients; however, it emphasises a middle-aged or older group that is disproportionately impacted by cardiac arrests.

### Location of OHCA

Table 1; Fig. 2 depict that during the pandemic, the proportion of OHCAs occurring at home grew from 84.73% in 2019 to 94.14% in 2020, then marginally decreased to 88.66% in 2021. During the epidemic, there was a significant increase (*p* < 0.001), which could be attributed to lockdowns, limited movement, and delayed medical care. Cases in public areas decreased to 3.85% during the First Wave from 12.30% in 2019 and somewhat rebounded to 9.21% in 2021. This tendency is consistent with lower outside activity and social constraints during the pandemic. OHCA cases in healthcare settings decreased slightly throughout the pandemic, from 2.49% in 2019 to 1.65% in 2020. This decrease could be attributed to fewer in-hospital care visits for non-COVID illnesses.

### Time of OHCA

Morning hours continuously accounted for the biggest share of OHCAs throughout all periods, slightly rising during the Second Wave (46.71% in 2021 vs. 44.15% in 2019), as shown in Fig. 2. Afternoon Hours: Afternoon OHCAs were consistent across all years, with roughly 28% of instances. Evening and late-night OHCAs gradually decreased from 27.49% (2019) to 25.21% (2021), as illustrated in Table 1; Fig. 2. The contemporary distribution of OHCAs indicates that pandemic-related pressure, changes in normal routines, and healthcare interruptions had no impact on cardiac arrest timing.

### Risk factors

**Fig. 3:**
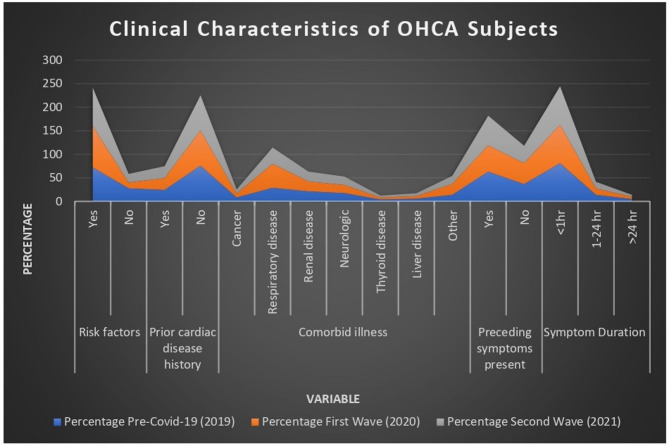
Clinical features of OHCA subjects during pre-COVID-19 and COVID-19 (1st and 2nd wave) outbreak groups

During the pandemic, there was a considerable increase in OHCA cases with identified risk factors. The statistically substantial increase (*p* < 0.001) can be attributed to the worsening of pre-existing conditions during the pandemic due to limited access to healthcare and delayed normal medical care. The data presented in Table 2; Fig. 3 show that the percentage of cases with no risk factors declined from 27.73% in 2019 to 12.25% in 2020, followed by a slight increase to 18.48% in 2021. The pandemic most likely resulted in underdiagnosis of asymptomatic or low-risk patients.

### Prior cardiac disease history

Cases with a prior heart illness remained stable across three periods (Table 2). Prior cardiac disease continued to contribute to OHCA despite the pandemic’s impact.

### Comorbid illness

#### Respiratory disease

During the pandemic, the prevalence of respiratory diseases ascended from 28.75% in 2019 to 50.97% in 2020, then decreased to 34.63% in 2021, as shown in Fig. 3. COVID-19 may have exacerbated respiratory problems, leading to this rise. Significant value was illustrated in Table 2. (*p* < 0.001).

#### Other comorbidities

Other comorbidities, such as renal illness, neurologic problems, thyroid disorders, and liver problems, had little variation over three periods. As reflected in Fig. 3, the percentage of “other” comorbidities increased from 14.06% in 2019 to 22.99% in 2020, indicating the impact of COVID-19 on overall health issues (*p* < 0.001), included in Table 2.

### Preceding symptoms

According to Fig. 3, the percentage of patients who presented with prior symptoms decreased during the First Wave (55.65%) compared to 2019 (63.26%), and then returned to pre-pandemic levels in 2021 (63.09%) (*p* < 0.001); significant value was illustrated in Table 2. Delays in seeking medical help, either out of fear of catching COVID-19 or due to barriers to healthcare access, may be the cause of this decline in symptomatic presentation in 2020.

### Symptom duration

Across all time periods, the majority of OHCA cases had symptoms for less than an hour (81.09%, 81.96%, and 81.42% in 2019, 2020, and 2021, respectively, as shown in Table 2; Fig. 3). There were few differences in cases with extended symptom durations (1–24 h or > 24 h), suggesting that the most frequent presentation of cardiac arrest was still acute symptom onset.

### OHCA witnessed

**Fig. 4:**
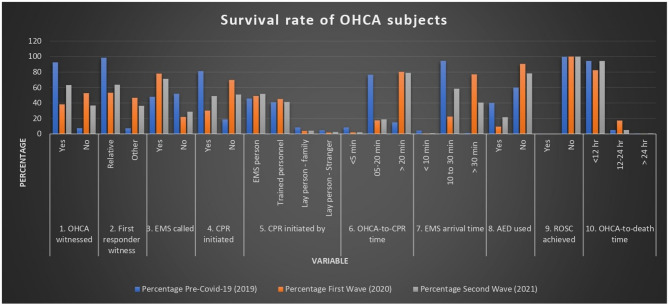
Survival rate of OHCA subjects after resuscitation attempt during pre-COVID-19 and COVID-19 (1st and 2nd wave) outbreak groups

Table 3; Fig. 4 show a significant decrease in the number of OHCAs witnessed (38.3%) due to the pandemic. This decrease, particularly during the First Wave, is likely due to pandemic-related variables such as increased social isolation and delayed emergency care.

### The first responder witnessed

During the pandemic, there was a large decrease in relatives functioning as first responders (53.26%). However, the share of “other” first responders grew from 7.29% in 2019 to 46.74% in 2020 (Table 3; Fig. 4), which could be due to decreased family interactions and increased reliance on external support.

### EMS calls and responses

Table 3 reflects that during the pandemic, the proportion of OHCAs when EMS was called in increased. EMS response times, however, substantially deteriorated. Responses under 10 min decreased from 4.36% in 2019 to barely 0.49% in 2020 and 0.93% in 2021. Responses lasting more than 30 min increased significantly, rising from 1.14% in 2019 to 77.04% in 2020 and 40.58% in 2021 (Fig. 4). This study demonstrates that the COVID-19 pandemic significantly influenced critical OHCA-related factors. During the pandemic waves, EMS response times increased substantially, with a greater proportion of cases experiencing delayed arrivals (> 30 min). This delay may have reduced the likelihood of successful resuscitation.

### CPR initiation

During the epidemic, CPR initiation fell abruptly: 81.2% in 2019, 30.19% in 2020, and 49.02% in 2021. There was a greater need for EMS workers to perform CPR. 45.79% in 2019, 49.09% in 2020, and 51.81% in 2021 (Table 3; Fig. 4). Layperson participation, especially among family members, declined dramatically, most likely as a result of insufficient training, anxiety about coming into contact with COVID-19, or unwillingness to administer CPR in a high-risk setting.

### Time interval between OHCA and CPR initiation

The data reflects there was a significant decrease in rapid CPR initiation (< 5 min): 8.42% in 2019, 2.12% in 2020, and 2.21% in 2021. There was a significant increase in delays (> 20 min) in starting CPR: 15% in 2019, 80.3% in 2020, and 78.92% in 2021 (Table 3). This highlights the significant lags in emergency response throughout the epidemic, which probably led to worse results (Fig. 4).

### AED/Defibrillator use

Table 3 shows that during the epidemic, AED use dramatically declined: 9.46% in 2020, 21.71% in 2021, and 40.08% in 2019. A lack of availability or a decline in community-level readiness may be the cause of the decrease in access.

### ROSC (Return of spontaneous Circulation)

ROSC remained exceedingly low throughout all time periods, with no survivors reported during the pandemic years (2020–2021) and only two reported prior to the pandemic (Table 3; Fig. 4). These findings indicate the consistently poor outcomes linked with OHCA, highlighting difficulties in emergency response and availability of advanced care both before and during the pandemic.

### Time interval between OHCA and death

Most fatalities happened within 12 h: 94.39% in 2019, 82.44% in 2020, and 94.3% in 2021, as reflected in Table 3. In 2020, Fig. 4shows a minimal increase in the 12- to 24-hour survival rate (17.36%), potentially due to postponed medical interventions.

The pandemic’s considerable influence on OHCA outcomes and reactions is highlighted by the significant *P*-values (*p* < 0.001) for all of the aforementioned aspects. Survival odds were significantly impacted by decreased bystander involvement, EMS response delays, and decreased use of AEDs and CPR. These results highlight the necessity of public health programs to bridge gaps in the emergency response system and enhance bystander readiness and willingness in times of crisis. All significant values are illustrated in Tables 1 and 2, and 3.

## Discussions

Researchers have extensively studied the OHCA trends and survival status of the COVID-19 pandemic. Several studies show OHCA cases markedly increased during the lockdown period, thought to be largely attributable to delayed emergency response and a decrease in bystander intervention. Marijon et al. (2020) described a significant increase in cases of OHCA in Paris, France, during the pandemic, underscoring the impact on emergency medical services (EMS) and hospitals (NEJM). Similarly, Baldi et al. found a surge in OHCA in Italy alongside a decrease in bystander CPR because of infection fears (European Heart Journal 2020). The prevalence of out-of-hospital cardiac arrest (OHCA) and its survival outcomes are among the most serious issues in this area, with some studies indicating that the survival rate of OHCA has been declining during the pandemic. Scquizzato et al. (2021) did a review of OHCA survival during COVID-19 and showed that survival rates were much lower than before the pandemic because of delays in emergency medical services and overwhelmed healthcare systems [34]. Additionally, Lim et al. (2022) from the Society for Cardiovascular Angiography & Interventions found in their study on OHCA trends before and after COVID-19 that there was a seasonal drop in the number of patients who regained spontaneous circulation (ROSC) and those who survived to leave the hospital. Bystander CPR and EMS response times also suffered [35]. Perkins et al. (2021) reported that fear of viral transmission reduced bystander CPR rates in a paper published in Circulation [36]. In a study by Awad et al. (2023) from a South Asian country near India, the number of patients who regained spontaneous circulation (ROSC) was much lower during the pandemic compared to before the pandemic [37]. The decrease in bystander CPR during the pandemic is also well-documented. A statistically significant reduction in bystander CPR was also detected over the different pandemic stages (during COVID-19 vs. pre-COVID) in a retrospective study published in the International Journal of Emergency Medicine by Awad et al. (2024), reinforcing the arrest response-damaging consequence of the pandemic [38]. This indicates that critical resuscitation outcomes may have been negatively impacted during the pandemic, potentially as a result of prolonged EMS response times, delayed recognition, reduced witnessed arrests, and lack of bystander-initiated CPR.

These findings are consistent with the observations from our study and highlight the role of enhancing pre-hospital care interventions in future pandemics. Including such studies will help to provide context and comparative data on trends in OHCA and survival rates pre- and post-pandemic, thereby reinforcing the public health implications of the findings observed.

In the current study, home-based OHCAs increased during the pandemic, suggesting that there must be improved emergency response times and public understanding of CPR and AED use in residential settings. The age and gender distributions remained consistent, indicating that the pandemic’s primary impact was logistical rather than demographic, with no evidence of heightened risk for specific groups. Decreased OHCAs in public areas reflected the effects of lockdowns and restricted movement on incident locations. These findings underscore the importance of robust emergency response procedures for home-based incidents. The time-of-day pattern for OHCAs remained stable, suggesting that physiological and lifestyle factors were more influential than pandemic-related disruptions. Strengthening community-based emergency care and expanding access to AEDs and CPR training, particularly in residential areas, is critical. The pandemic significantly altered the distribution of OHCAs, while demographic and temporal trends remained largely unchanged. Addressing these shifts requires a targeted public health approach to improve home-based cardiac arrest outcomes. During the First Wave, comorbid respiratory disorders and risk factors increased, highlighting the need for comprehensive management of pre-existing conditions during public health emergencies. The rapid onset of symptoms (within an hour) stresses the importance of timely emergency response systems. Public health initiatives should focus on enhancing bystander CPR training and AED accessibility. The decline in symptomatic presentations in 2020 indicates barriers to seeking care during the pandemic, emphasising the need for public education on identifying and responding to cardiac crises [39].

The pandemic brought attention to the need for chronic illness management to reduce OHCA risks. Addressing these issues can help healthcare systems better prepare for future emergencies, minimising the pandemic’s impact on cardiac care. The statistics reveal significant delays in both EMS response and CPR initiation during the pandemic, which likely increased mortality rates. Bystander-initiated CPR and AED use declined drastically, underscoring the need for defibrillator accessibility and widespread CPR training [40]. The strain on healthcare services during the pandemic is evident in prolonged EMS response times [41]. Future emergency preparedness programs should prioritise increasing EMS capacity during crises. The near-universal mortality and lack of ROSC during the pandemic demonstrate the importance of prompt action in OHCA cases [42]. Investing in public health campaigns to promote CPR and AED awareness is essential. The pandemic exposed vulnerabilities in OHCA management, including delayed access to healthcare, overwhelmed EMS services, and inadequate bystander intervention, all of which negatively impacted survival rates [43].

## Conclusions

Overall, there was a crucial and heterogeneous impact of the COVID-19 pandemic on incidences, management, and outcomes of OHCA in patients. Not only do the longer emergency response times and lack of bystander involvement increase the number of OHCA events, but the shift was also associated with worse neurological outcomes and lower rates of survival for many patients. Even though the change in resuscitation guidelines was necessary for provider safety, it significantly increased the difficulty of emergency care. These challenges demonstrate the importance of robust public health policies, increased community CPR training, and resource planning to sustain emergency care in the event of medical emergencies in the future. These learnings related to the pandemic provide opportunities to reinforce emergency preparedness, alternative response protocols for OHCA, and preservation of cardiac emergency therapy during anticipated future public health cataclysms.

### Implications

- Enhancing public health campaigns about CPR and AED use.
- Offering ongoing intensive care in times of emergencies.
- Lessons from the pandemic that could be adapted for future emergency measures.

### Limitations

This study has several limitations. First, the absence of a centralised data collection system and a national OHCA registry system. Second, we did not perform multivariable analysis because the dataset did not meet the required assumptions for reliable modelling. The above findings are based on univariate statistical comparisons, which may not fully account for potential confounding factors influencing the interactions between variables and could affect the generalisability of the conclusions. We recommend further studies with larger sample sizes or multivariable modelling to validate these findings and account for confounding factors.

#### Variable definitions

The following variables were defined and used in the analysis:

##### EMS called

Indicates whether emergency medical services were called when OHCA event occurred. Indicated “Yes” if at least one formal EMS provider (government or private ambulance service) was called, regardless of whether EMS arrived. “No” if the patient was transported by private means without EMS contact.

##### Dead on arrival

Patients who presented to the emergency department without vital signs and were pronounced deceased soon after hospital assessment.

##### ROSC (Return of spontaneous Circulation)

Clinically defined as a palpable pulse and effective blood circulation achieved by resuscitation practice as documented by the treating emergency physician.

##### Bystander CPR

Cardiopulmonary resuscitation performed by a layperson, non-medical individual or bystander before the emergency responders or hospital staff arrives.

##### Witnessed arrest

An arrest that was observed directly by a bystander or health care provider at the time the event occurred.

##### Trained personnel

Refers to individuals who have received formal training in basic life support (BLS) or first aid (e.g., paramedics, nurses, doctors, medical students, individuals certified in CPR, or certified EMS staff) who were present at the scene and involved in prehospital care, patient transport, or at the emergency department.

##### Mode of transport

Method by which the patient arrived at the emergency department (EMS ambulance, private ambulance, private vehicle, other).

##### Time to hospital arrival

The duration minutes (from estimated time of cardiac arrest to time of registration at the emergency department).

##### ACLS

Advanced Cardiovascular Life Support.

## Data Availability

The datasets used and/or analyzed during the current study are available from the corresponding author on reasonable request.
